# Induction of a chloracne phenotype in an epidermal equivalent model by 2,3,7,8-tetrachlorodibenzo-*p*-dioxin (TCDD) is dependent on aryl hydrocarbon receptor activation and is not reproduced by aryl hydrocarbon receptor knock down

**DOI:** 10.1016/j.jdermsci.2013.09.001

**Published:** 2014-01

**Authors:** Alison R. Forrester, Martina S. Elias, Emma L. Woodward, Mark Graham, Faith M. Williams, Nick J. Reynolds

**Affiliations:** a Dermatological Sciences, 2nd Floor Leech Building, Medical Science, Institute of Cellular Medicine, Newcastle University, NE2 4HH, United Kingdom; b Toxicology, Institute of Cellular Medicine, Newcastle University, NE1 7RU, United Kingdom; c Global Safety Assessment, Alderley Park, AstraZeneca, Cheshire, United Kingdom

**Keywords:** AhR, aryl hydrocarbon receptor, AHRR, aryl hydrocarbon repressor protein, α-NF, α-naphthoflavone, β-NF, β-naphthoflavone, CYP1A1, cytochrome P450 1A1, ITE, 2-(1′H-indole-3′-carbonyl)-thiazole-4-carboxylic acid methyl ester, TCDD, 2,3,7,8-tetrachlorodibenzo-*p*-dioxin, TGM-1, transglutaminase-1, XRE, xenobiotic response element, Aryl hydrocarbon receptor, TCDD, β-Naphthoflavone, ITE, Keratinocyte, Epidermal equivalent

## Abstract

**Background:**

2,3,7,8-Tetrachlorodibenzo-*p*-dioxin (TCDD) is a potent activator of the aryl hydrocarbon receptor (AhR) and causes chloracne in humans. The pathogenesis and role of AhR in chloracne remains incompletely understood.

**Objective:**

To elucidate the mechanisms contributing to the development of the chloracne-like phenotype in a human epidermal equivalent model and identify potential biomarkers.

**Methods:**

Using primary normal human epidermal keratinocytes (NHEK), we studied AhR activation by XRE-luciferase, AhR degradation and CYP1A1 induction. We treated epidermal equivalents with high affinity TCDD or two non-chloracnegens: β-naphthoflavone (β-NF) and 2-(1′H-indole-3′-carbonyl)-thiazole-4-carboxylic acid methyl ester (ITE). Using Western blotting and immunochemistry for filaggrin (FLG), involucrin (INV) and transglutaminase-1 (TGM-1), we compared the effects of the ligands on keratinocyte differentiation and development of the chloracne-like phenotype by H&E.

**Results:**

In NHEKs, activation of an XRE-luciferase and CYP1A1 protein induction correlated with ligand binding affinity: TCDD > β-NF > ITE. AhR degradation was induced by all ligands. In epidermal equivalents, TCDD induced a chloracne-like phenotype, whereas β-NF or ITE did not. All three ligands induced involucrin and TGM-1 protein expression in epidermal equivalents whereas FLG protein expression decreased following treatment with TCDD and β-NF. Inhibition of AhR by α-NF blocked TCDD-induced AhR activation in NHEKs and blocked phenotypic changes in epidermal equivalents; however, AhR knock down did not reproduce the phenotype.

**Conclusion:**

Ligand-induced CYP1A1 and AhR degradation did not correlate with their chloracnegenic potential, indicating that neither CYP1A1 nor AhR are suitable biomarkers. Mechanistic studies showed that the TCDD-induced chloracne-like phenotype depends on AhR activation whereas AhR knock down did not appear sufficient to induce the phenotype.

## 1 Introduction

The aryl hydrocarbon receptor (AhR) is a highly conserved member of the basic helix-loop-helix PER/ARNT/SIM family of transcription factors. It is activated by a wide range of planar polycyclic aromatic hydrocarbons (PAH) or halogenated aromatic hydrocarbons (HAH), including the highly potent ligand 2,3,7,8-tetrachlorodibenzo-*p*-dioxin (TCDD). AhR is known to mediate a wide range of chemical toxicity in animals and humans [1], [2], [3]. Exposure to AhR ligands, mainly TCDD, has been linked to a range of human toxicities which were particularly evident following industrial accidents [4], [5], [6]. In humans, the most obvious toxicity caused by TCDD is a severe skin acneform condition known as chloracne [7], [8], [9], with the most famous case being former Ukrainian President Victor Yushchenko [10].

Chloracne is characterised by two histological hallmarks: loss of sebaceous glands and presence of epidermal cysts or comedones [11], [12]. Within the follicle wall, the open basket-weave pattern of stratum corneum is lost and becomes thicker and compact resulting in a follicular plug and the characteristic comedone observed in involved skin [8], [11], [13], [14]. The mechanisms underlying this phenotype however, have not yet been fully characterised. Because of significant differences between rodent and human skin and the lack of suitable animal models, 3-D human skin equivalent models have been evaluated for the further study of chloracne pathogenesis. Although these models are not currently advanced enough to contain structures such as sebaceous glands, nor do they contain the potential to develop cysts, TCDD-treated epidermal equivalents have been shown to develop key features found in chloracne histology including: (1) hyperkeratinisation of the stratum corneum and (2) a thinner viable cell layer [15]. In vivo, these characteristics both appear within the follicle wall [8], [11], [13], [14], highlighting the potential of human 3-D models for the study and elucidation of underlying pathogenic mechanisms. In addition, during drug development, screening for chloracnegenic potential of novel compounds is performed but current assays are based on CYP1A1 induction in non-keratinocyte cell lines.

While a causal relationship between exposure to TCDD and chloracne is established, other AhR agonists such as β-NF, do not appear to induce chloracne in humans and β-NF does not induce chloracnegenic-associated changes in keratinocytes [16]. Structurally, AhR ligands are typically planar hydrocarbons which may be divided into halogenated or polycyclic aromatic hydrocarbons (HAH or PAH respectively). HAHs (such as TCDD) can have AhR binding affinities roughly 1000-fold higher than PAHs, reviewed in [17], [18]. As the majority of AhR studies to date have focused on the effects of xenobiotic compounds on the AhR, in this study we opted to include a physiological AhR ligand, 2-(1′H-indole-3′-carbonyl)-thiazole-4-carboxylic acid methyl ester (ITE), which is a tryptophan derivative known to regulate responses in a variety of cell types [19], [20], for comparison with AhR ligands TCDD and β-NF. ITE was originally isolated from porcine lung [21] and is a potent AhR ligand. In primary mouse lung fibroblasts it has been shown to elicit AhR-dependent responses at 0.2 μM comparable to those elicited by 0.2 nM TCDD [22]. Its potency is roughly 2–4 orders of magnitude less than TCDD in whole cell assays in a mouse hepatoma cell line (depending on endpoint measured) [23] and it has been shown to be 5 times more potent than β-NF [21]. Competitive binding studies showed that AhR binding affinities of β-NF and ITE were of a similar magnitude whereas the AhR binding affinity of TCDD was higher (*k*_i_ for murine AhR:ITE = 3 nM, β-NF = 2 nM, TCDD = 0.5 nM) [21]. ITE has been shown to induce AhR degradation in primary mouse lung fibroblasts which recovers after roughly 24 h, consistent with its rapid removal by metabolism [22], [23].

Although TCDD is a potent AhR ligand it remains unclear whether cutaneous toxicity induced by TCDD is mediated by AhR. Interestingly, AhR null mice exhibit a skin phenotype which has similarities to chloracne including changes in the hair follicle [2], suggesting that development of chloracne may be dependent on a lack of AhR rather than a result of AhR activation causing up-regulation of the AhR gene battery. These data may be relevant to the pathophysiology of chloracne because AhR degradation occurs following agonist-induced AhR activation [24], [25]. However, mouse skin differs significantly from human skin, re-enforcing the need for further studies in models relevant to human skin.

AhR activity has been shown to increase as keratinocytes differentiate [26], [27], [28] suggesting that monolayer keratinocytes alone are not an ideal model for studying chloracnegenic effects. 3-D epidermal equivalent models allow keratinocytes to differentiate forming discrete viable cell layers and a fully differentiated stratum corneum [29] and provide an environment to maintain stem cells [30], thereby enabling their long-term culture. Epidermal stem cells are also hypothesised to be one of the main cell types involved in chloracne development [31]. This model therefore allows the direct study of pharmacological or agonist treatment on cell proliferation, differentiation and death, in a physiologically relevant context. In epidermal equivalents, TCDD has been previously shown to induce aberrant and early onset of differentiation and a phenotype consisting of a thin viable cell layer, hyperkeratosis and stratum corneum thickening [15], [32].

In this study we have utilised normal human epidermal keratinocytes (NHEKs), an epidermal equivalent model of chloracne and a combined pharmacological and genetic approach to gain further mechanistic insight into chloracne pathophysiology. We hypothesised that activation of the AhR would regulate the chloracne-like phenotype in the epidermal equivalent model. By comparing the effects of chloracnegenic and non-chloracnegenic AhR ligands on AhR activation and on the phenotype and differentiation of keratinocytes in an epidermal equivalent model, we show that the chloracne-like phenotype depends on AhR activation whereas AhR knock down is not sufficient to induce the phenotype.

## 2 Materials and methods

### 2.1 Primary cell and epidermal equivalent culture

Primary NHEKs were isolated from redundant skin samples, following approval by the local ethical committee and informed consent from the patient. Primary NHEKs were maintained in Epilife (Lonza, Basel, Switzerland) supplemented with 60 μM CaCl_2_, penicillin/streptomycin (1%) and Human Keratinocyte Growth Supplement (Lonza) at 37 °C with 5% CO_2_ in a humidified atmosphere, as described [33]. Cells were passaged at ∼70% confluency and used from passage 1–4.

Three dimensional epidermal equivalents were formed by seeding primary NHEKs at high confluency (maximum passage 4) on to polycarbonate cell culture inserts (0.4 μm pore; Millicell, Merck KGaA, Darmstadt, Germany) in Epilife supplemented with 1.5 mM CaCl_2_ (as described [33]) and grown at the air/liquid interface in medium supplemented with 5 μg/ml Vitamin C for 5 days. The epidermal equivalents were then treated for a further 7 days, formalin fixed and embedded in paraffin or lysed for protein or RNA extraction.

### 2.2 Chemicals and treatment regimens

AhR ligands were purchased from Sigma–Aldrich (Dorset, UK): TCDD (48599), β-NF (N-3633), ITE (I-9283) and α-NF (N-5757). AhR antagonist, 2-methyl-2H-pyrazole-3-carboxylic acid-(2-methyl-4-*o*-tolyl-azophenyl)-amide (CH-223191) was purchased from Calbiochem, Merck KGaA. Stock solutions were prepared in dimethyl sulfoxide (DMSO) and protected from light. Use of plastics was minimised and cultures were treated with fresh medium containing compound every 48 h.

### 2.3 Luciferase assays

AhR activation was measured using an AhR-dependent luciferase reporter (pXRE4-SV40-Luc) [34]. Primary NHEKs were co-transfected using TransIT Keratinocyte transfection reagent (E7-0084; Geneflow, Staffordshire, UK) according to manufacturer's instructions, with a renilla luciferase construct control vector (pRLTK; Promega, Wisconsin, USA) to which firefly values were normalised. Cells were treated with ligand for 48 h and assayed using the Dual luciferase reporter system (E1910; Promega), as previously described [33], [35].

### 2.4 Western blot analysis

Primary NHEKs were treated with vehicle (DMSO), TCDD, β-NF or ITE. Monolayer cell lysis was performed in RIPA buffer [35], epidermal equivalents were lysed in urea buffer: 8 M Urea, 50 mM Tris (pH 8), 5 mM EDTA, 2.5% SDS, 1 mM sodium fluoride, 1 mM PMSF, 1 mM sodium orthovanadate, plus a protease inhibitor cocktail.

Primary antibodies used were CYP1A1 (sc-25304; SantaCruz Biotechnology, Santa Cruz, CA, USA), AhR (MA1-514; Affinity Bioreagents, NY, USA), filaggrin and involucrin (Novocastra, Milton Keynes, UK) or TGM-1 (Biomedical Technologies, Madrid, Spain). Equal protein loading was confirmed with monoclonal β-actin (A 5316; Sigma–Aldrich) or GAPDH antibody (MAB 374; Merck Millipore, Dormstadt Germany) and anti-mouse IgG peroxidise conjugate secondary antibody (PI 2000; Vector Laboratories, Peterborough, UK) Membranes were imaged using a Fujifilm FLA-3000 fluorescence image analyser (Fujifilm, Dusseldorf, Germany). Bands were analysed according to densitometry and presented as a ratio of CYP1A1 or AhR:β-actin normalised to vehicle using Multigauge V2.2 software (Fujifilm).

### 2.5 Histological analysis of epidermal equivalents

Paraffin embedded sections were stained with haematoxylin and eosin. Images were taken on a Zeiss Axioimager light microscope.

For immunochemical analysis, the following antibodies were used: AhR (SC-5579; SantaCruz) involucrin (NCL-INV, Novocastra), filaggrin (FLG01, MS-449-P1; Lab Vision Neomarkers, MI, USA), or transglutaminase-1 (BT-621; Biomedical Technologies) with 488-conjugated alexafluor anti-rabbit or anti-mouse secondary antibodies and To-Pro 3 nuclear stain (Life Technologies, Paisley, UK). Images were captured using a Leica confocal inverted TC II SP2 system (Leica Microsystems, Germany), as described [35]. An isotype control was included in each experimental and gain and offset were set to this and kept constant throughout each imaging set. Images shown for FLG and INV staining represent an overlay of three z stacks to ensure clear representation of basal, upper viable and cornified layers of the epidermal equivalent. This is referred to hereafter as “sum of mid z”. Images were processed using Adobe Photoshop 6.0 (Adobe Systems Europe Ltd., Berkshire, UK) maintaining equal colour settings. Quantification of AhR intensity was performed in Volocity 6.1 (PerkinElmer, Cambridge, UK).

### 2.6 AhR knock down

#### 2.6.1 Packaging and production of the shRNA vector in 293T cells

HEK293T cells were cultured in complete DMEM containing 10% foetal calf serum. GIPZ lentiviral shRNAmir vectors were purchased from Open Biosystems (CO, USA) with distinct sequences targeting AhR (RHS4531, individual clones referred to as 1382 and 2320), empty GFP (RHS4349, EGFP) or non-silencing (RHS4346, NS) control sequences. 293T cells were co-transfected with pMD2.G envelope plasmid (5 μg), pCMVδ8.91 packaging plasmid (15 μg) and pGIPZ shRNA transfer plasmid (20 μg) using the calcium chloride precipitation method as previously described [17], [18].

#### 2.6.2 Transduction of primary NHEKs with lentiviral shRNA

At 70–80% confluence, primary NHEKs were spin transduced in a DMEM (plus 10% FCS) solution containing 25% viral particles with 4 μg/ml polybrene and grown in selection medium (1 μg/ml puromycin in complete Epilife) for 5 days. Transduced NHEKs were then grown to form epidermal equivalents as described previously in Materials and Methods and harvested in parallel for H&E analysis or Western blotting. Lentiviral work was carried out in accordance with safety requirements from the Health and Safety Executive.

### 2.7 Measurement of CYP1A1 gene expression by real time-qPCR

Total RNA was extracted from epidermal equivalents using an RNeasy micro kit (Qiagen) following the manufacturer's instructions and reverse transcribed using Superscript VILO Mastermix (Life Technologies). Quantitative PCR was performed using the validated Fam conjugated TaqMan gene expression assay for CYP1A1 (Hs00153120_m1, Life Technologies) with GAPDH (Life Technologies) as a housekeeping control. For AhR, a validated exon-spanning probe based assay was used with 18S as a housekeeping gene: forward primer 5′–3′CGAATGGCTCATTAAATCAGTTATGG, reverse primer 5′–3′TATTAGCTCTAGAATTACCACAGTTATCC (Integrated DNA Technologies Inc, UK) [36]. Samples were run in technical duplicates using a Gene Amplification PCR System 9700 (Applied Biosystems).

### 2.8 Statistical analysis

#### 2.8.1 Luciferase assay

**Fig. 1:**
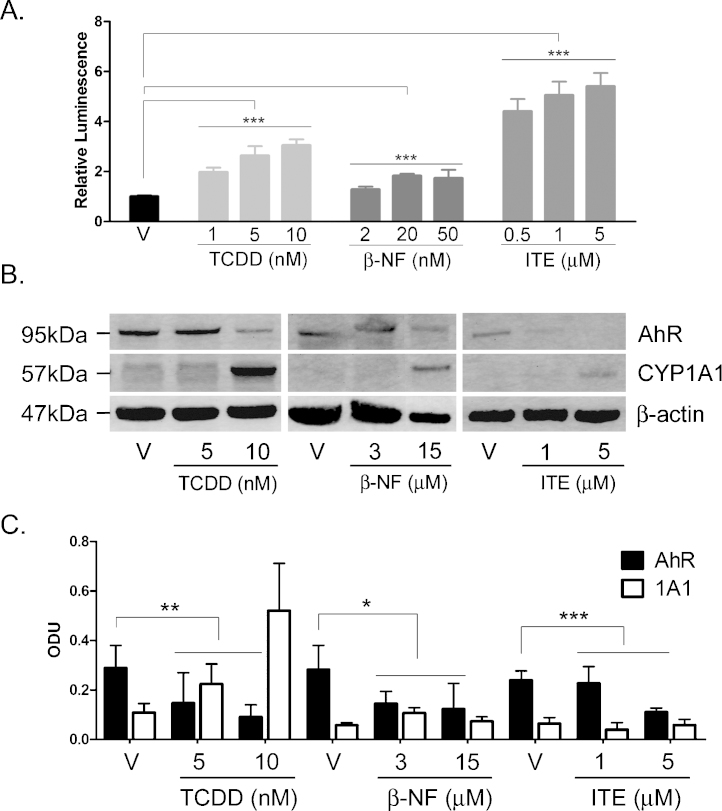
TCDD, β-NF and ITE induce AhR activation in NHEK, (A) Primary normal human epidermal keratinocytes (NHEKs) were co-transfected with XRE4-SV40-luciferase and renilla-luciferase constructs and treated with vehicle, TCDD, β-NF or ITE as indicated for 48 h. XRE-luciferase activity was measured and firefly:renilla luciferase ratio was normalised to vehicle. Data shown are mean ± sem, *n* = 9 (triplicate wells from 3 donors). Analysis of trend comparing vehicle to ligand, ***P < 0.0004. (B) Primary NHEKs were treated every 48 h for 8 days (on days 0, 2, 4 and 6) with vehicle or ligand as indicated. Samples were lysed and Western blotting performed. (C) Densitometry was carried out on blots probed with antibodies against AhR and CYP1A1 and normalised to β-actin. Densitometry represents mean ± sem from 3 donors; graph shows results of one-way ANOVA. Effects of TCDD on AhR: one-way ANOVA, **P = 0.007, analysis of trend *P* = 0.02. Effects of β-NF on AhR: one-way ANOVA, *P = 0.02, analysis of trend, NS. Effects of ITE on AhR: one-way ANOVA, ***P = 0.0002, analysis of trend *P* < 0.02.

Significant effects of ligand treatment were analysed using one-way ANOVA to compare vehicle to ligand treatment (Fig. 1 and Supplementary Figure 4) or ligand treatment alone to ligand plus α-NF (Fig. 6) with Dunnett's post hoc test or analysis of trend to indicate dose dependency.

#### 2.8.2 Western blotting

One-way ANOVA was used to compare vehicle to ligand treated samples at each time-point. Two-way ANOVA was used to compare ligand treatment and time-point (Fig. 1) or ligand treatment to α-NF and time (Fig. 6 and Supplementary data, Figures 1–3).

#### 2.8.3 RT-qPCR and quantification of viable cell layer thickness

One-way ANOVA was used to compare vehicle to ligand treated samples with Dunnett's post hoc test. If one-way ANOVA was significant, post hoc tests were performed and indicated in figure legends.

#### 2.8.4 RT-qPCR for AhR

*t*-Test was used to compare EGFP to 1382 or 2320. Significance is equal to *P* < 0.05. Statistical analysis was performed using GraphPad Prism 5 (GraphPad, San Diego, CA, USA).

## 3 Results

### 3.1 Activation of the AhR by TCDD, β-NF and ITE in primary NHEKs

In order to determine the effects of the chloracnegenic AhR agonist TCDD and the non-chloracnegenic AhR agonists β-NF and ITE on AhR activation in NHEKs and to determine dosimetry for use in epidermal equivalents, transcriptional activation by XRE-luciferase assay, CYP1A1 induction and AhR degradation were initially studied in primary NHEK monolayer cultures.

TCDD, β-NF and ITE induced concentration dependent transcriptional activation of the AhR reporter gene after 48 h exposure in NHEKs (Fig. 1A) (one-way ANOVA *P* < 0.0001, analysis of trend *P* < 0.0004). A preliminary time course study showed induction of XRE-luciferase at 24 h, 48 h and 96 h. 48 h was selected for study as this was the earliest time point at which reproducible and consistent effects were observed (data not shown). The concentrations of ligand required for significant transcriptional activation varied 1000-fold between ligands, consistent with different affinities for the AhR. Based on molarity TCDD was most potent, followed by β-NF and ITE (relative luminescence = 5, 1.5 and 1 respectively). Luciferase assays were performed with concentrations of β-NF in the nM range. This was necessary because high concentrations (2 μM and above) of β-NF quenched the XRE-luciferase activity, as previously reported by Wang [37]. However, as nM concentrations of β-NF had little phenotypic effect and failed to robustly induce CYP1A1 in keratinocyte monolayers, Western blotting experiments were performed using higher concentrations (3 and 15 μM) of β-NF. These showed CYP1A1 induction and AhR degradation consistent with AhR activation by 3 and 5 μM β-NF (Fig. 1B).

Western blot analysis of NHEKs treated every 48 h for 8 days, showed that compared to vehicle, TCDD (5 or 10 nM) induced greater than 75% AhR degradation (*P* = 0.007, analysis of trend: *P* = 0.02) while CYP1A1 was induced in a dose dependent manner to 4-fold (Fig. 1B and C and supplementary Figure 1). Treatment with β-NF (3 or 15 μM) resulted in ∼50% AhR degradation (*P* = 0.02, analysis of trend: NS) but inconsistently induced CYP1A1 expression up to 2-fold but neither of the changes were statistically significant (Fig. 1B and C and Supplementary Figure 2). The Western blot in Fig. 1B is part of a complete time course shown in its entirety in Supplementary Figure 2. ITE (1 or 5 μM) induced AhR degradation by greater than 40% (*P* = 0.0002, analysis of trend: *P* < 0.02) but induced little expression of CYP1A1 (Fig. 1B and C and Supplementary Figure 3). Both basal and induced expression of CYP1A1 varied between donors and therefore densitometry was performed on blots from 3 independent donors and normalised to their own vehicle treated controls (Fig. 1C). To ensure that CYP1A1 levels had not been induced earlier than 48 h and returned to basal levels by the time of harvesting, we additionally tested time points 24 h post treatment with TCDD, β-NF or ITE; however no significant CYP1A1 protein induction was detected (data not shown). In the repeated dosing (every 48 h) experiments conducted in NHEK monolayer cultures, up regulation of CYP1A1 protein expression was only robustly detected after 8 days of TCDD exposure but AhR degradation (as a consequence of AhR activation) was detected from day 2 by all ligands.

In summary, all ligands degraded AhR from an early time point (Supplementary Figures 1–3) but only TCDD robustly induced CYP1A1, although all ligands showed the capacity to induce CYP1A1 in a donor dependent manner.

### 3.2 TCDD induced a chloracne-like phenotype in epidermal equivalents

**Fig. 2:**
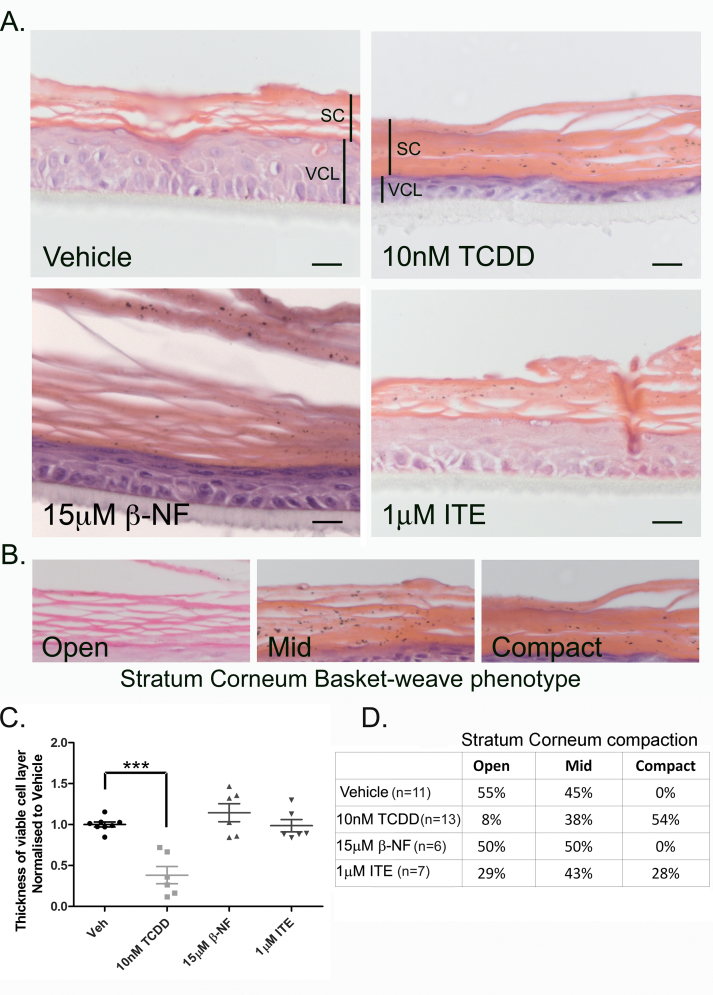
TCDD caused decreased thickness of the viable cell layer and compaction of the stratum corneum in the epidermal equivalent model. (A) Epidermal equivalents were grown as described in materials and methods and treated with vehicle, TCDD, β-NF or ITE every 48 h for 7 days. After 7 days, equivalents were fixed, paraffin embedded and stained with H&E. (A) viable cell layer (VCL) and stratum corneum (SC) are marked by labelled black lines. Images are representative of effects in 3 donors. Scale bar = 20 μm. (B) Basket-weave formation of the stratum corneum of each section were characterised as open, mid way or compact. (C) Using Image J, 6 measurements of the viable cell layer were taken from 2 images per treatment for each donor. Individual values and mean (±sem) are shown for 3 independent donors. Dunnett's post hoc test compared vehicle to ligand, TCDD: ***P < 0.0001. (D) Compaction of the stratum corneum for each treatment per donor were characterised as demonstrated in (B). Values represent percentage of sections analysed from a minimum of 6 donors.

To compare the phenotypic effects of different AhR ligands, we treated epidermal equivalents with TCDD, β-NF or ITE every 48 h for 7 days. In TCDD treated epidermal equivalents, the histological phenotype, consisting of a thin viable cell layer and a thicker and compacted stratum corneum with parakeratosis (Fig. 2), showed strong resemblance to changes observed within vellus follicles and comedones in chloracne [8], [11], [13]. The chloracne phenotype was quantified using the two parameters: viable cell layer thickness and stratum corneum compaction (Fig. 2B–D). After treatment with 10 nM TCDD for 7 days, the stratum corneum became compacted and thicker compared to vehicle, with 54% of the samples exhibiting a completely compact phenotype, only 8% exhibiting the open basket-weave phenotype and others mid compaction (based on basket-weave characterisation as described in Fig. 2B). Stratum corneum compaction in β-NF treated samples did not differ from control, with both vehicle and β-NF treated equivalents exhibiting a combination of open and mid compacted stratum corneum phenotypes. Following treatment with 1 μM ITE 29% of epidermal equivalents were classified as open phenotypes, 43% were classified as mid and 28% were classified as compacted (Fig. 2A and D). This suggests that at this concentration, ITE interacted with the mechanisms leading to stratum corneum compaction but to a lesser extent than TCDD, while β-NF did not induce stratum corneum compaction. Lower doses of ITE were tested in this system and resulted in no phenotypic effects (data not shown).

The viable cell layer in TCDD treated samples was significantly thinner than in vehicle treated samples (one-way ANOVA with Dunnett's post hoc test *P* < 0.0001). Notably this significant reduction in the thickness occurred solely in TCDD treated epidermal equivalents and not in β-NF or ITE treated samples (Fig. 2A and C). Parakeratosis, indicating disrupted differentiation was often present in TCDD, β-NF and ITE treated epidermal equivalents. Consistent with induction of apoptosis-independent cell death by TCDD [38], we also failed to detect caspase 3 activation (data not shown).

**Fig. 3:**
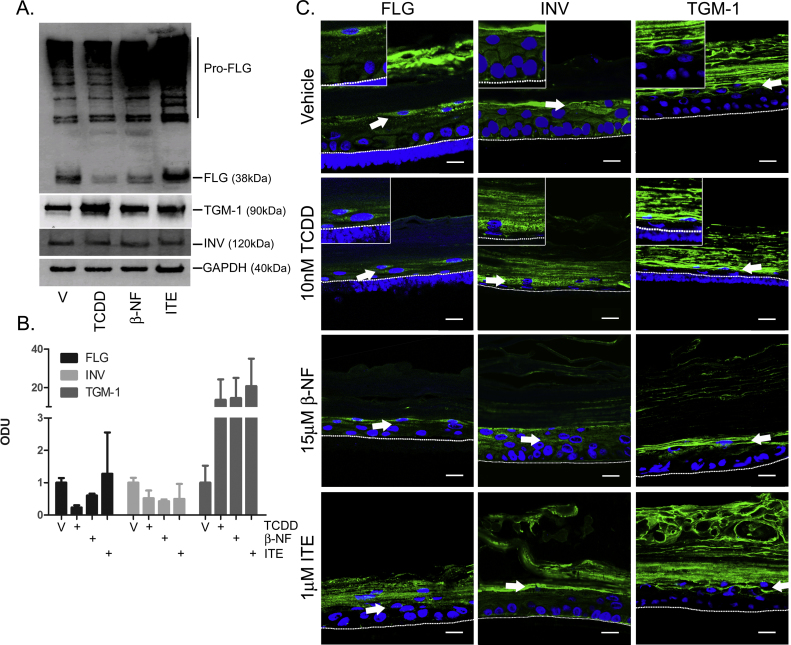
AhR activation induces dysregulated expression of involucrin, filaggrin and transglutaminase-1. Epidermal equivalents were grown and treated with vehicle, 10 nM TCDD, 15 μM β-NF or 1 μM ITE every 48 h for 7 days. (A) Western blotting was performed and blots were probed with antibodies against filaggrin (FLG), involucrin (INV) and transglutaminase-1 (TGM-1), with GAPDH as loading control. (B) Densitometry was carried out on blots probed with antibodies against FLG, INV and TGM-1 and normalised to GAPDH. Densitometry represents mean (±sem) from 2 donors. (C) Immunochemistry was performed using antibodies against FLG (left column), INV (centre column) or TGM-1 (right column) with Oregon green (488) tagged secondary antibody and To-pro-3 (blue) nuclear stain. Mid z section (TGM-1) and sum of 3 mid z (FLG, INV) images were captured by confocal microscopy and are representative of epidermal equivalents from 3 donors. Inserts show images at higher magnification (115×). Dotted white lines represent junction between basal layer and polycarbonate membrane and white arrows indicate points of interest. Scale bars = 20 μm.

To investigate the mechanism of thinning of the viable cell layer and the involvement of altered cellular differentiation in the observed phenotype, expression of the differentiation markers filaggrin, involucrin and TGM-1 were measured by Western blot (Fig. 3A). TCDD and β-NF down-regulated filaggrin expression while ITE had little effect. Levels of involucrin expression remained unaffected following AhR ligand treatment. On average from Western blots quantified from three donors, TGM-1 protein expression was increased by all three ligands (Fig. 3A and B). However TCDD induced consistent up-regulation of TGM-1 in all three donors studied, while β-NF and ITE only induced TGM-1 in some donors.

To investigate changes to the localisation of filaggrin, involucrin and TGM-1 within the keratinocyte layers undergoing differentiation, immunochemistry was performed. All ligands induced aberrant changes in expression of involucrin and filaggrin to different degrees. Involucrin was expressed in closer proximity to the basal cell layer in response to TCDD and β-NF. Consistent with Western blot data, filaggrin expression was reduced by TCDD and β-NF and to a lesser extent by ITE. Filaggrin puncta were also decreased in number and became unevenly distributed throughout the granular cell layer and in cell layers in closer proximity to the basal layer, particularly in response to TCDD. TGM-1 was expressed on the inner edge of the spinous and granular cells in vehicle controls. In TCDD-treated epidermal equivalents, TGM-1 expression appeared increased in spinous and granular layers and its localisation was less evenly distributed around the cell membrane, while β-NF and ITE induced little change (white arrows, Fig. 3C). Together, these data indicate that TCDD induced aberrant and early onset of differentiation in primary NHEKs most consistently, with β-NF and ITE inducing early differentiation to a lesser extent in the spinous and granular layers.

### 3.3 TCDD, β-NF and ITE induce AhR activation in epidermal equivalents, but the chloracne-like phenotype is not a result of AhR degradation

**Fig. 4:**
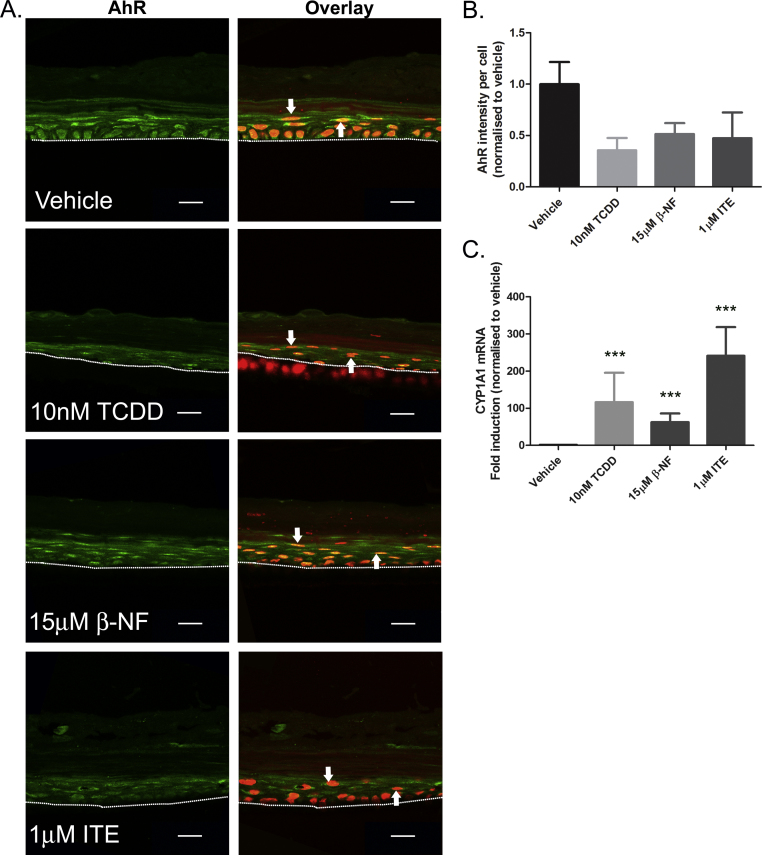
TCDD, β-NF and ITE induce AhR activation in epidermal equivalents. Epidermal equivalents were grown and treated with vehicle, 10 nM TCDD, 15 μM β-NF or 1 μM ITE every 48 h for 7 days. (A) Immunochemistry was performed using an antibody against AhR, Oregon green (488) tagged secondary antibody and To-pro-3 (red) nuclear stain. Mid z sections were captured by confocal microscopy and some of 3 mid-sections are shown. Dotted white lines represent polycarbonate membrane and white arrows indicate points of interest. Scale bars = 20 μm. (B) Intensity of AhR fluorescence within the viable cell layer was quantified in Volocity software and normalised to number of nuclei present within the region of interest. Graph represents quantification from 2 donors. (C) mRNA was isolated from ligand treated epidermal equivalents and relative expression measured by RT-qPCR. Dunnett's post hoc test compared vehicle to ligand, ***P < 0.0001. Error bars represent mean (±sem) from 3 donors.

To verify AhR activation by TCDD, β-NF and ITE in epidermal equivalents, protein expression and pattern of AhR expression were determined by immunochemistry (Fig. 4A) and AhR expression was quantified by measuring green fluorescence intensity in the viable cell layer, normalised to number of nuclei present (Fig. 4B). In vehicle treated samples, AhR was mainly localised in the nuclei of basal cells, with low levels of cytoplasmic staining, while in the suprabasal layers cytoplasmic AhR staining was evident. This pattern of localisation was consistent across donors (white arrows, Fig. 4A). Although nuclear translocation of AhR in keratinocytes may be induced by AhR agonists including TCDD and coal tar [27], [39], transcriptional regulation is tightly regulated. Thus, AhR nuclear localisation does not necessarily identify cells in which AhR is bound to promoters and inducing transcriptional activation. TCDD treatment over 7 days decreased basal nuclear AhR staining, consistent with AhR down-regulation following activation. β-NF and ITE also induced down-regulation of AhR in the basal layer (Fig. 4A and B).

As a robust measurement of AhR transcriptional activation in epidermal equivalents, we utilised RT-qPCR to measure levels of CYP1A1 after 7 days of repeated treatment of epidermal equivalents with vehicle, TCDD, β-NF or ITE (Fig. 4C). Expression of CYP1A1 mRNA was significantly increased by all ligands, analysed by one-way ANOVA (*P* < 0.0001), indicating the presence of AhR activation in epidermal equivalents and confirming the ability of all ligands tested to induce CYP1A1 in this model.

**Fig. 5:**
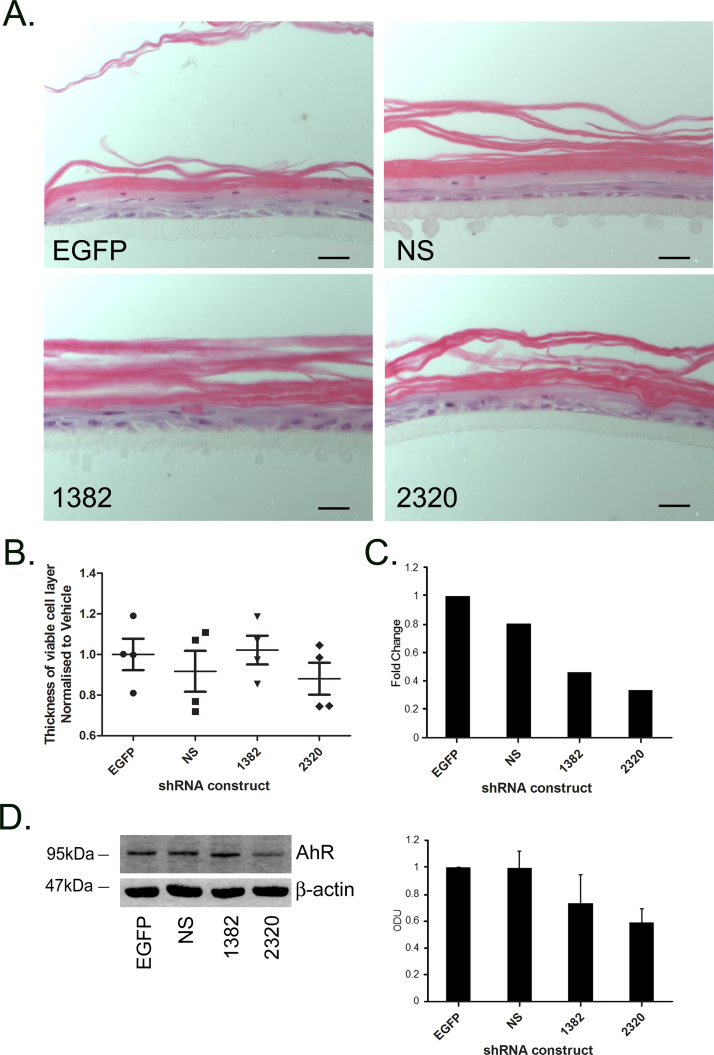
Development of the chloracne phenotype is not a direct result of AhR down-regulation. (A) Primary NHEKs were transduced with lentiviral shRNA constructs against AhR (1382, 2320), empty GFP (EGFP) or non-silencing (NS) control sequences and used to form epidermal equivalents. Samples were harvested, paraffin embedded and H&E stained. Scale bars = 20 μm. (B) 6 measurements of the viable cell layer were taken from 2 images per treatment for each donor. Individual values and mean (±sem) are shown. (C) mRNA was isolated from NHEK monolayers in parallel with epidermal equivalent cultures and knock down of AhR measured by RT-qPCR and normalised to 18S. Results presented as fold change relative to cells transfected with NS vector (NS). *t*-Test compared EGFP to 1382 (not significant) or 2320 (**P < 0.01) bars represent mean values from 2 donors. (D) AhR knock down epidermal equivalents were harvested in RIPA buffer and Western blotting performed. Blots were probed with antibodies against AhR and loading control β-actin. Images are representative of 3 donors. (E) Densitometry was carried out on blots probed with antibodies against AhR and normalised to β-actin. Results presented as fold change relative to cells transfected with empty GFP vector (note alternate order of bars). Densitometry represents mean (±sem) from 3 donors.

Currently it is unknown whether AhR-dependent toxicity is a consequence of down-regulation of AhR or up-regulation of AhR regulated genes by AhR activation, or a combination thereof. In order to study the potential role of reduced AhR expression, we used lentiviral shRNA constructs to knock down AhR in epidermal equivalents and determined the consequent effect on the phenotype (Fig. 5). Fig. 5A shows H&E staining of AhR knock down epidermal equivalents compared to empty GFP and non silencing control epidermal equivalents. AhR knock down in NHEKs (utilised for seeding of epidermal equivalents) was verified by RT-qPCR (Fig. 5C) and Western blotting (Fig. 5D and E). Construct 2320 induced consistent knock down across donors, inducing on average ∼60% knock down by RT-qPCR and on average ∼40% by Western blotting (*P* ≤ 0.01) which was comparable to the effects of TCDD on AhR protein levels in NHEKs (Fig. 1B and C). A second shRNA construct against AhR (1382) was less effective, inducing on average ∼50% knock down by RT-qPCR (Fig. 5C) and ∼20% knock down by Western blot (Fig. 5E), and was variable across donors. AhR protein expression was unaffected following transduction of empty GFP and non-silencing control constructs by Western blot (Fig. 5D and E), although by RT-qPCR empty GFP transfection induced a small degree of AhR down regulation (Fig. 5C). For this reason we normalised fold changes of AhR expression to the non silencing control in RT-qPCR experiments.

Using the same parameters as used earlier in this paper to measure presence of the chloracne phenotype (stratum corneum compaction (Fig. 5A) and decreased viable cell layer thickness (Fig. 5B)), we observed that AhR knock down did not induce any chloracne-like changes in epidermal equivalents (Fig. 5A and B).

These data suggest that down-regulation of AhR per se is insufficient to induce the chloracniform phenotypic changes observed in the epidermal equivalent model.

### 3.4 α-NF blocks TCDD-induced chloracne-like phenotype

**Fig. 6:**
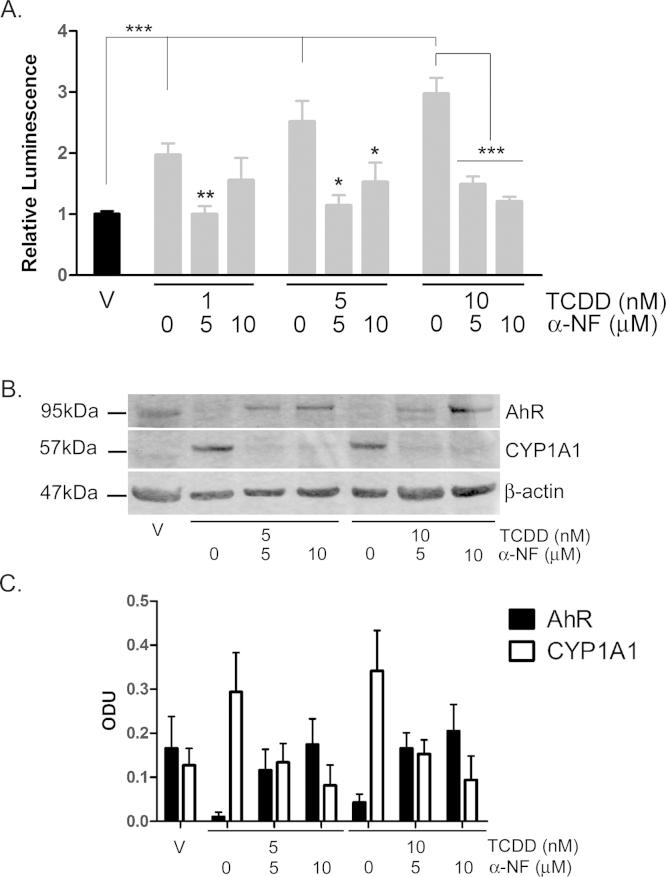
TCDD-induced AhR activation is inhibited by α-NF in primary NHEKs. (A) Primary NHEKs were co-transfected with XRE4-SV40-luciferase and renilla-luciferase control constructs and treated with vehicle or TCDD and/or α-NF as indicated for 48 h. Luciferase activity was measured and the firefly:renilla luciferase ratio was normalised to vehicle. Data shown are mean (±sem), *n* = 9 (triplicate wells from 3 donors). Two-way ANOVA: post hoc tests comparing vehicle to TCDD ± α-NF, Dunnett's: **/*, analysis of trend: ***P < 0.0001. (B) Primary NHEKs were treated with vehicle, TCDD and/or α-NF every 48 h as indicated for 8 days. Cells were lysed and proteins separated by Western blotting. Blots were probed with AhR and CYP1A1 antibodies with β-actin as loading control. Western blot is representative of duplicate blots from 3 donors. (C) Densitometry was performed on blots from 3 donors probed with antibodies against AhR (black bars) and CYP1A1 (white bars) and normalised to β-actin. Two-way ANOVA and analysis of trend: effects of TCDD on AhR, *P = 0.02. Densitometry is represents mean (±sem) from 3 donors.

To further investigate the AhR dependence of the effects of TCDD in NHEKs, we inhibited ligand-induced AhR activation with α-NF [40], [41]. α-NF alone (30 nM–24 μM) induced low levels of AhR transcriptional activation at 48 h, although this was not significant (one-way ANOVA, Supplementary Figure 4). α-NF has been shown in previous studies to elicit the most effective inhibition of ligand-induced AhR activation at concentrations that also *induce* some degree of AhR activation [40]. Therefore, despite low AhR activation induced by α-NF (Supplementary Figure 4), we used 5 and 10 μM α-NF at 8 days to measure inhibition of TCDD induced AhR activation (Fig. 6).

α-NF (5 and 10 μM) significantly inhibited TCDD-induced AhR transcriptional activation (*P* < 0.001) (Fig. 6A) as well as TCDD-induced AhR degradation and CYP1A1 induction, most markedly at later time points, day 8 (Fig. 6B and C).

Together, this data is consistent with partial agonist activity of α-NF and indicates that alone α-NF may induce low AhR activation. However, when added in combination with a potent AhR agonist such as TCDD, the data clearly demonstrate that α-NF acts as an AhR antagonist and inhibits agonist-induced AhR activation in NHEKs.

**Fig. 7:**
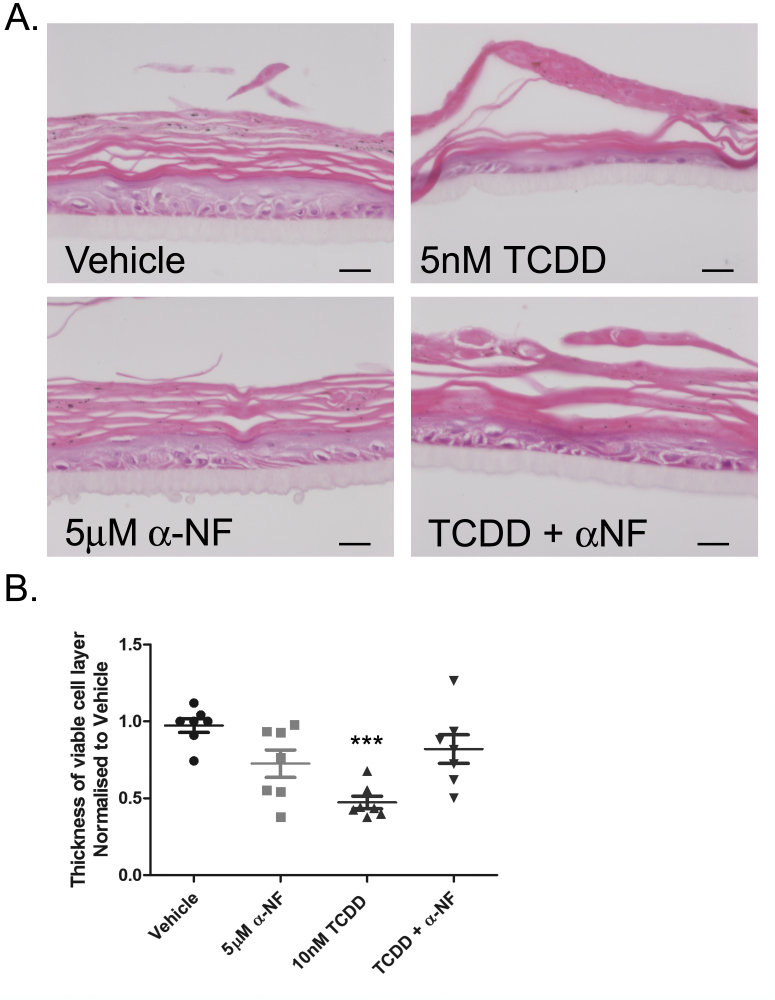
Inhibition of AhR activation by α-NF partially blocks TCDD-induced phenotype in epidermal equivalents. (A) Epidermal equivalents were grown and treated with vehicle, 10 nM TCDD and/or 5 μM α-NF every 48 h for 7 days. After 7 days, equivalents were fixed, embedded in paraffin and stained with H&E. (B) Using Image J, 6 measurements of the viable cells layer were taken from 2 images per treatment for each donor. One-way ANOVA: Dunnett's post hoc test comparing vehicle to ligand, ***P = 0.0004. Individual values and mean (±sem) are shown for 3 donors.

To define the role of transcriptional AhR activation in the development of the chloracne-like phenotype, epidermal equivalents were co-treated with 10 nM TCDD and 5 μM α-NF. α-NF partially blocked the development of the TCDD-induced phenotype (Fig. 7A), resulting in less thinning of the viable cell layer (one-way ANOVA, *P* = 0.0004) and partially reinstated the open basket-weave phenotype of the stratum corneum. α-NF alone caused a slight decrease in thickness of the viable cell layer, corresponding with the low levels of AhR activation shown in Supplementary Figure 4. However, this was not statistically significant and importantly, the thickness of the viable cell layer in TCDD and α-NF co-treated epidermal equivalents was not significantly different to vehicle (Fig. 7B). This data indicates that α-NF acted as an AhR antagonist in epidermal equivalents and inhibited the TCDD-induced chloracne phenotype.

In summary, α-NF blocked TCDD-dependent AhR degradation and CYP1A1 induction in monolayer NHEKs. Co-treatment of TCDD and α-NF in epidermal equivalents partially reinstated the normal phenotype.

## 4 Discussion

Chloracne is a recognised human toxicity of TCDD but the dependence on AhR and the relationship between AhR transcriptional activation and AhR down-regulation in the pathogenesis of chloracne remains ill-defined. In this study, we compared the effects of potent chloracnegen TCDD (which has high affinity for the AhR and high potency), the non-chloracnegen β-NF (which has a low affinity for the AhR and low potency), and non-chloracnegen and endogenous compound ITE (a highly potent AhR agonist [42]) in NHEKs and an epidermal equivalent model. The main findings of this study are that: (1) neither CYP1A1 induction nor AhR degradation by AhR agonists in NHEKs correlated with the ligand-induced chloracne phenotype in the epidermal equivalent model or documented chloracne potential, (2) the TCDD-induced chloracne-like phenotype in epidermal equivalents was blocked by pharmacological inhibition (by α-NF) of AhR transcriptional activation and is therefore AhR dependent and (3) development of the chloracne phenotype is a result of AhR activation, not AhR down-regulation as demonstrated by the lack of phenotypic changes induced by AhR knock down in epidermal equivalents.

When treated with TCDD, the epidermal equivalent model used in this study robustly exhibited the two main characteristic phenotypes of chloracne observed in vellus follicles: (1) a thickened and compacted stratum corneum and (2) a thin viable cell layer [8], [11], [13]. Therefore, our data underscores the utility of this model for studying the underlying pathogenic mechanisms of chloracne. As demonstrated in this paper, monolayer NHEKs are useful for studying pathway activation including AhR, but a more physiologically relevant model allows extrapolation to disease phenotypes and delineation of effects on cellular differentiation in a 3-D context. AhR activation has not previously been defined in the epidermal equivalent model. By immunofluorescence, we observed that AhR showed nuclear localisation with epidermal equivalents, predominantly within the basal layer. To the best of our knowledge, localisation of AhR protein has not been previously reported within skin or epidermis. AhR agonists, including TCDD and coal tar have previously been shown to induce nuclear localisation of AhR in keratinocytes [27], [39]. Additionally, loss of cell–cell contact and signalling through E-cadherin may also regulate AhR nuclear localisation in keratinocytes [43], [44]. The functional significance of nuclear AhR within basal keratinocytes remains to be explored but nuclear localisation of AhR does not necessarily equate to transcriptional activation driven by AhR. For example, the negative regulatory AhR repressor protein (AHRR) competitively inhibits transcription driven by AhR activation [34], [45]. AHRR is itself induced by AhR and provides a negative feedback loop; thus the relative levels and localisation of AhR and AHRR may be critical to the regulation of down-stream signalling. On the other hand, following nuclear translocation and transcriptional activation, AhR undergoes degradation (Fig. 1) [25], [43] and this does provide a readout of recent AhR activation. Consistent with this, AhR immunofluorescence within the basal nuclei was decreased by AhR agonist treatment and most substantially by TCDD (Fig. 4A and B).

The full chloracne-like phenotype was induced only by TCDD, although ITE partially induced the chloracne-like phenotype, as shown by induction of partial stratum corneum compaction in the epidermal equivalent model. This may be attributed to the relatively high concentrations of ITE that we used in these experiments, which are higher than the low basal levels expected to be found physiologically [21]. At nM concentrations, ITE had no phenotypic effect in the epidermal equivalent model.

In monolayer NHEKs, all ligands showed the capacity to up-regulate CYP1A1 protein (see Supplementary Figures 1–3) although CYP1A1 protein was only robustly up-regulated by TCDD. We can be confident that the CYP1A1 induction did not occur at an earlier time point because of three reasons: (1) samples were tested for CYP1A1 induction 24 h post treatment in addition to 48 h post treatment, (2) in HepG2s, that have a higher metabolic capacity than keratinocytes [46], half life of CYP1A1 protein is roughly 9 h [47] and therefore would not have decreased completely by 24 h and (3) as previously published by Du et al. [16] and in agreement with our data, CYP1A1 was only robustly induced by TCDD after 8 days treatment [16]. In contrast, CYP1A1 mRNA was clearly induced by all ligands in the epidermal equivalent model, however this most likely reflects the high sensitivity of RT-qPCR compared to Western blotting. The induction of CYP1A1 by TCDD at 8 days may be a result of TCDD accumulation within the cell which is a known characteristic of TCDD (reviewed in [48]). This is an important differential characteristic between TCDD and the non-chloracnegenic ligands tested in this study. However, these results may also reflect the increased endogenous activity of AhR activation in more differentiated cells [16], or be a result of relatively low levels of endogenous CYP1A1 protein in keratinocytes compared to hepatocytes [46], [49], [50].

We observed inter-individual variation in levels of basal and induced CYP1A1 protein detected by Western blotting (Fig. 1B and C and Supplementary Figures 1–3) and in qRT-PCR (Fig. 4C). We controlled for this by normalising data to their own untreated controls and utilising multiple (at least 3) donors in each experiment. Inter-individual variation may be due to AhR polymorphisms within the population [51] or potentially to previous exposure to ultraviolet radiation [52] or exogenous AhR agonists such as benzo[a]pyrene that is found in cigarette smoke [53]. However, the latter are less likely to be relevant as keratinocytes studied were derived from normal skin (usually discarded from operations on sun-protected sites). AhR polymorphisms are known to affect levels of induced CYP1A1 within the population, as described by Smart and Daly [51] in a study of a similar geographical population to our study. They reported a 103-fold variation in induced CYP1A1 in human lymphocytes which significantly correlated with two polymorphisms in the AhR [51]. In line with previous studies showing induction of CYP1A1 protein by β-NF and ITE in human keratinocytes and mouse lung fibroblasts [22], [49], we can conclude that although CYP1A1 is a marker of AhR activation and correlated with ligand potency in our studies, it is not a specific biomarker of chloracnegenic potential.

Metabolism of TCDD is known to be low [54] (a factor that contributes to the persistence of chloracne [7]) but inducible by AhR activation [10], [55], while the metabolism of β-NF is known to be more rapid, through cytochromes P450 1A1 and 1A2 [56], [57]. Xenobiotic metabolising cytochrome P450 enzymes in keratinocytes and in human epidermal equivalents are of low activity [46] and with the multiple dosing regimen used in these studies the concentrations of ligands would have been maintained.

α-NF inhibited TCDD-induced AhR activation and chloracne-like phenotype in epidermal equivalents, providing evidence that TCDD-induced effects in NHEKs are AhR-dependent and that AhR activation itself, not AhR down-regulation, is responsible for the chloracne-like phenotype. This is further supported by the observation that a chloracne phenotype was not induced by AhR knock down in the epidermal equivalent model. Although α-NF is known to exhibit both agonistic and antagonistic activity, it is a well characterised partial AhR agonist [41], [58]. For example, α-NF has been shown to induce AhR-XRE complex formation in HepG2s from 1 μM, while at the same concentration it begins to inhibit formation of the AhR-XRE complex induced by 2 nM TCDD [58]. As we have shown in NHEKs, XRE-luciferase activity was induced at low levels from roughly 120 nM to 12 μM, while 5 and 10 μM α-NF robustly inhibited TCDD induced AhR activation (Supplementary Figure 4). Together with studies in a variety of cell types [41], [58] and further publications in NHEKs which utilise concentration ranges of α-NF spanning those used in this study [56], [59] we can be confident that our results reflect antagonist activity of α-NF. In recent years, new AhR antagonists have emerged which are claimed to exert no agonist activity. However, we have shown that in NHEKs, one such compound CH-223191 [60] activates XRE-luciferase at concentrations of 3 μM and above (Supplementary Figure 4B), similar to α-NF.

Early onset of terminal differentiation induced by TCDD in keratinocytes and epidermal equivalents has been previously reported in the literature [15], [16], [32], [61], [62]. Consistent with these findings, we found that filaggrin and involucrin expression were altered by TCDD and β-NF. New findings in this paper indicate that ITE also influenced filaggrin and involucrin expression. A factor contributing to the regulation of keratinocyte differentiation by AhR may be the recently identified AhR dependent regulation of filaggrin by an upstream XRE domain [62]. Additionally, Du et al. [16] described that in human keratinocytes TCDD induced expression and activation of TGM-1, while β-NF did not. However, in contrast to their findings, we have shown by Western blot that β-NF also up-regulates TGM-1 expression, and additionally that ITE can also induce TGM-1, although there was more donor variation in response to β-NF and ITE than TCDD. Together, these data are consistent with early onset of terminal differentiation being a contributory mechanism to the development of the chloracne phenotype. However, the changes in filaggrin, involucrin or TGM-1 expression did not appear specific to TCDD and they did not correlate with chloracne potential.

The MAPK-ERK pathway is involved in epidermal homeostasis [63] and has been proposed as a mechanism of chloracne lesion development. Previous studies have shown activation of EGFR/c-src/MAPK/c-Fos pathways by AhR agonists [64], [65] but reports vary [66] and robust and complete AhR-induced activation of the pathway remains to be demonstrated. Interestingly in studies of biopsies taken from chloracne lesions of patients putatively exposed to dioxins, Tang et al. [67] showed up regulation of c-Fos and Liu et al. showed an increase in pEGFR and p-MAPK [67], [68]. However, nowadays TCDD-induced chloracne is extremely rare and in recent case studies, exposure to a “cocktail” of compounds hinders interpretation of the pathophysiological role of TCDD and AhR activation. Although not the subject of our study, epidermal equivalent models allow specific putative pathways to be studied in the context of controlled chemical exposure and defined AhR activation. It would therefore be of interest to study the activation of EGFR/MAPK/c-Fos and how this relates to phenotype development.

In conclusion, the data presented in this paper further underscore the utility of epidermal equivalents for the study of chloracne pathogenesis and indicate that the development of the chloracne phenotype in epidermal equivalents in response to AhR agonists appears to be a result of AhR activation rather than AhR down regulation. However, neither CYP1A1 induction nor AhR degradation appear to be specific biomarkers for the chloracnegenic potential of compounds.

## Source of funding

This work was a CASE studentship funded by AstraZeneca and BBSRC.

## Figures and Tables

**Supplementary Fig. I:**
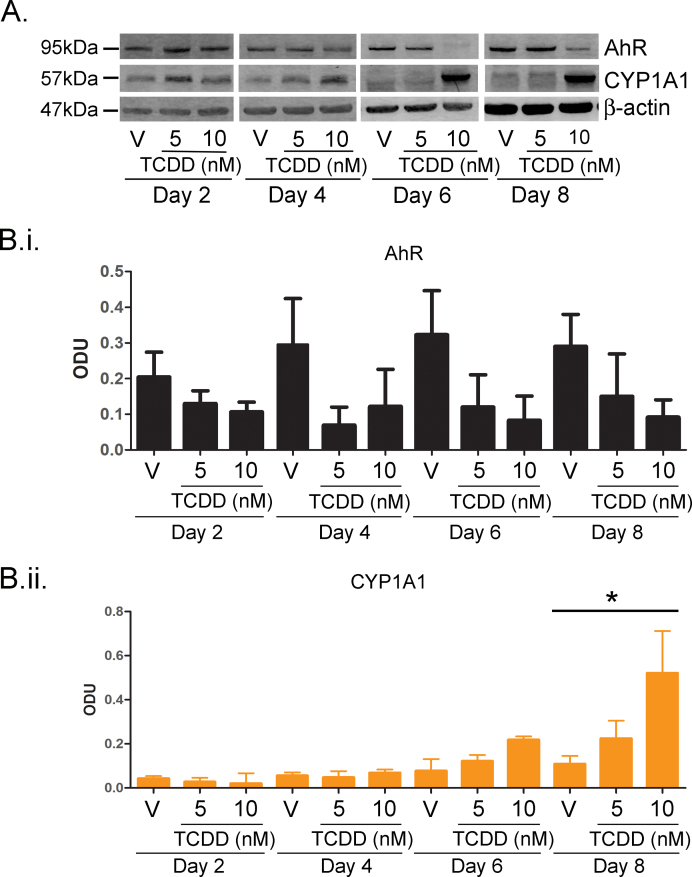
TCDD resulted in time and dose dependent AhR degradation and CYP1A1 induction between 2 and 8 days. (A) Primary NHEKs were treated for up to 8 days with vehicle, 5 or 10 nM TCDD as indicated. Samples were lysed and Western blotting performed. Densitometry was carried out on blots probed with antibodies against (B.i) AhR and (B.ii) CYP1A1 and normalised to β-actin. Densitometry represents mean ± sem from 3 donors. Two-way ANOVA: analysis of trend on effects of TCDD on AhR: *P = 0.02. Effects of time and TCDD on CYP1A1 induction, analysis of trend: **P < 0.002.

**Supplementary Fig. II:**
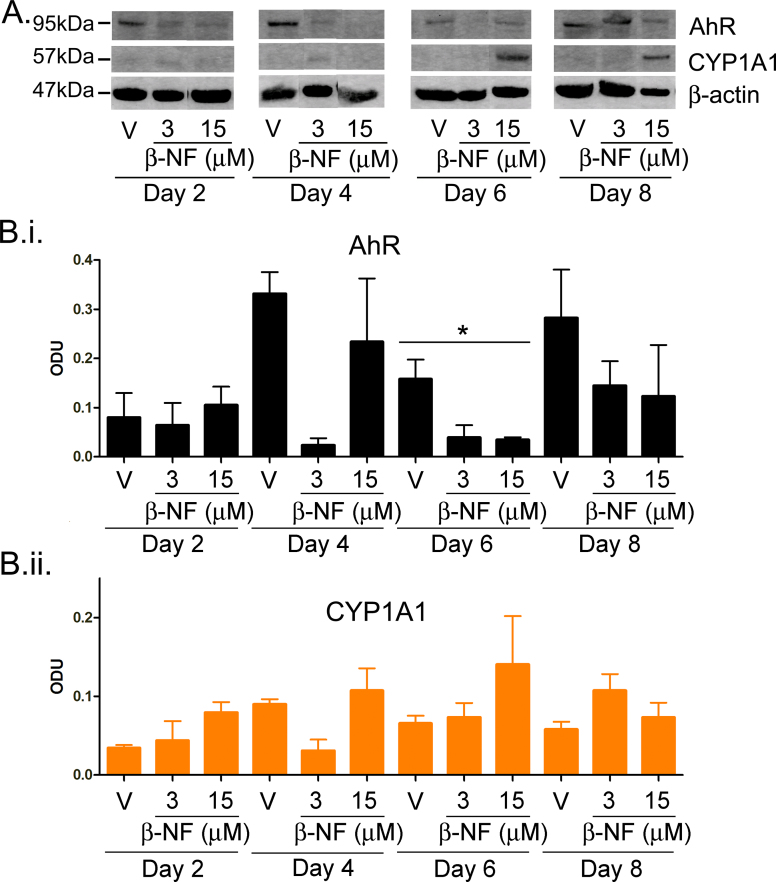
β-NF induced CYP1A1 and AhR degradation in primary NHEKs. (A) Primary NHEKs were treated for up to 8 days with vehicle or doses of β-NF as indicated. Samples were lysed and Western blotting performed. Densitometry was carried out on blots probed with antibodies against (B.i) AhR and (B.ii) CYP1A1 normalised to β-actin. Densitometry represents mean ± sem from 3 donors. Two-way ANOVA: analysis of trend on effects of β-NF on AhR, *P < 0.02. CYP1A1 induction was not significant.

**Supplementary Fig. III:**
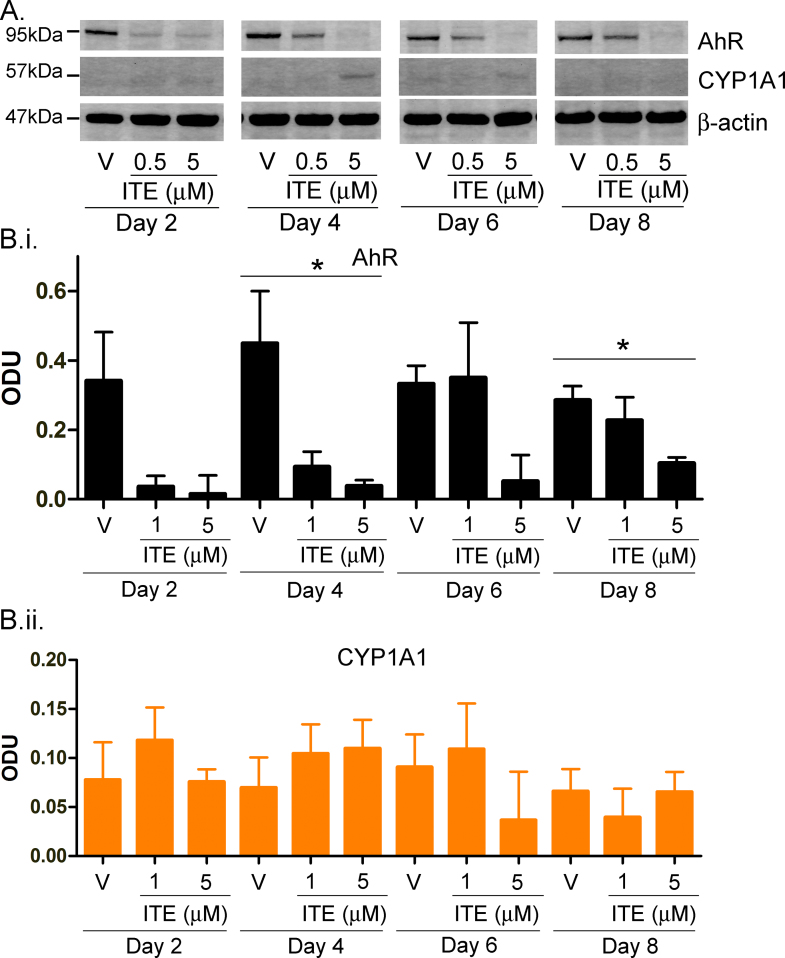
ITE induced CYP1A1 and AhR degradation in primary NHEKs. (A) Primary NHEKs were treated for up to 8 days with vehicle or doses of ITE as indicated. Samples were lysed and Western blotting performed. Densitometry was carried out on blots probed with antibodies against (B.i) AhR or (B.ii) CYP1A1 normalised to β-actin. Densitometry represents mean ± sem from 3 donors. Two-way ANOVA: analysis of trend on effects of ITE on AhR, *P < 0.02. CYP1A1 induction was not significant.

**Supplementary Fig. IV:**
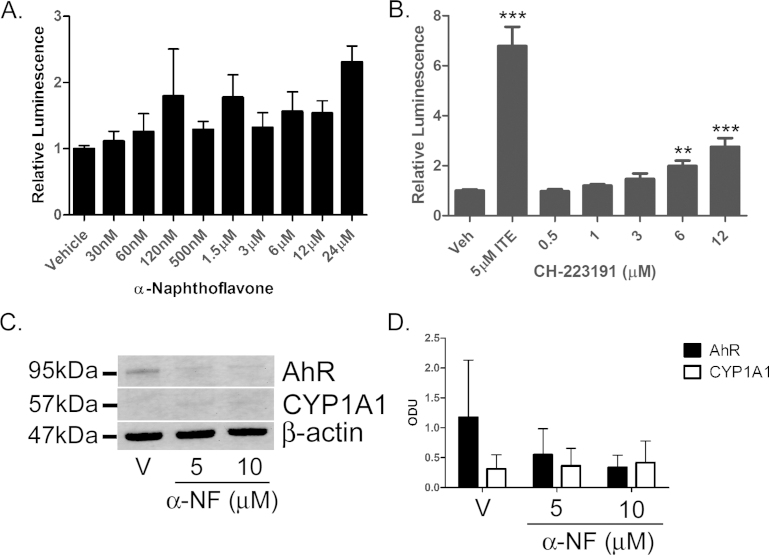
AhR agonist activity of α-NF and CH-223191. (A/B) Primary NHEKs were co-transfected with XRE4-SV40-luciferase and renilla-luciferase control constructs and treated as indicated for 48h. Luciferase activity was measured and the mean firefly:renilla luciferase ratio was normalised to vehicle ± sem. *n* = 9, triplicate wells from 3 donors. Effects of α-NF on XRE-luciferase by one-way ANOVA: NS. Effects of CH-223191 on XRE-luciferase assay by one-way ANOVA: *P* < 0.0001, Dunnett's post hoc test, **/*** as indicated 5 μM ITE included as positive control, note different scale bars between (A) and (B). (C) Primary NHEKs were treated for 8 days with vehicle or α-NF as indicated. Samples were lysed and Western blotting performed. (D) Densitometry was carried out on blots probed with antibodies against AhR and CYP1A1 and normalised to β-actin. Densitometry represents mean ± sem from 3 donors. Two-way ANOVA was performed comparing vehicle to α-NF and time. α-NF significantly induced AhR degradation (*P* < 0.05), CYP1A1 was not significantly induced.
